# Capillary nano-immunoassays: advancing quantitative proteomics analysis, biomarker assessment, and molecular diagnostics

**DOI:** 10.1186/s12967-015-0537-6

**Published:** 2015-06-06

**Authors:** Jin-Qiu Chen, Lalage M Wakefield, David J Goldstein

**Affiliations:** Collaborative Protein Technology Resource, Center for Cancer Research, National Cancer Institute, National Institutes of Health, 9000 Rockville Pike, Building 37, Room 2140, Bethesda, MD 20892 USA; Laboratory of Cancer Biology and Genetics, Center for Cancer Research, National Cancer Institute, National Institutes of Health, Bethesda, MD 20892 USA; Office of Science and Technology Resources, Center for Cancer Research, National Cancer Institute, National Institutes of Health, Bethesda, MD 20892 USA

**Keywords:** Capillary nano-immunoassay, Simple western, Cell signaling, Biomarker, Proteomics, Molecular diagnostic

## Abstract

There is an emerging demand for the use of molecular profiling to facilitate biomarker identification and development, and to stratify patients for more efficient treatment decisions with reduced adverse effects. In the past decade, great strides have been made to advance genomic, transcriptomic and proteomic approaches to address these demands. While there has been much progress with these large scale approaches, profiling at the protein level still faces challenges due to limitations in clinical sample size, poor reproducibility, unreliable quantitation, and lack of assay robustness. A novel automated capillary nano-immunoassay (CNIA) technology has been developed. This technology offers precise and accurate measurement of proteins and their post-translational modifications using either charge-based or size-based separation formats. The system not only uses ultralow nanogram levels of protein but also allows multi-analyte analysis using a parallel single-analyte format for increased sensitivity and specificity. The high sensitivity and excellent reproducibility of this technology make it particularly powerful for analysis of clinical samples. Furthermore, the system can distinguish and detect specific protein post-translational modifications that conventional Western blot and other immunoassays cannot easily capture. This review will summarize and evaluate the latest progress to optimize the CNIA system for comprehensive, quantitative protein and signaling event characterization. It will also discuss how the technology has been successfully applied in both discovery research and clinical studies, for signaling pathway dissection, proteomic biomarker assessment, targeted treatment evaluation and quantitative proteomic analysis. Lastly, a comparison of this novel system with other conventional immuno-assay platforms is performed.

## Background

Advances in the molecular analysis of genes, proteins and metabolites have greatly improved our understanding of biological processes and disease, and have increased our ability to monitor treatment response and stratify patients to improve treatment efficacy. Precision medicine facilitated by companion diagnostics is one of the driving forces accelerating the drug development process and improving therapeutic management [1]. For example, targeting HER2 over-expression for breast cancer treatment led to the development of Herceptin (trastuzumab, Genentech), the first approved monoclonal antibody drug [2]. Another well known example is the use of a K-ras mutation test as a predictor of poor response to EGFR inhibitor treatment [3].

To enable this type of molecular profiling, concerted effort has been put forth in both academic and industry sectors to develop and validate new technologies. In the past decade, many genome and transcriptome profiling technologies, including next-generation sequencing, single-nucleotide polymorphism (SNP), fluorescent in situ hybridization (FISH), and mRNA quantitation technologies, have been developed and successfully applied in areas from discovery and translational research to clinical practice [4–8]. However, DNA sequence information is not sufficient to predict the biological function of a protein, and gene expression does not always correlate with pathway activation level [9, 10]. Based on UniProt, there is no experimental evidence at the protein level for about 38% out of 20,000 protein-coding human genes [11]. In a study of correlation between protein and mRNA abundance in yeast [12], Gygi et al. found that for some genes, while the mRNA levels were about  the same, the protein levels varied by more than 20-fold. Conversely, invariant steady-state levels of certain proteins were observed with respective mRNA transcript levels that varied by as much as 30-fold. Post-translation modifications (PTM) of proteins, such as phosphorylation, acetylation and glycosylation, can alter a protein’s activity and affect disease development and treatment response [13–15]. Thus it is critical to directly measure proteins and their activation status to confirm genomic predictions and monitor treatment responses.

In contrast to major advances in DNA and RNA profiling, the investigation of signaling molecules at the protein level still faces many challenges, such as limited clinical sample size, poor reproducibility, unreliable quantitation, and difficulties in protocol standardization across different laboratories [16, 17]. There is a strong need to develop automated, high-throughput proteomic technologies for precise and accurate determination of protein levels and analysis of protein activation status, to facilitate both discovery research and clinical practice.

In this manuscript, we review the performance and applications of a novel capillary nano-immunoassay (CNIA) system (The Simple Western System™, marketed by ProteinSimple, CA, USA), a fully automated capillary electrophoresis system for characterization of proteins and their post-translational modifications. This new system overcomes many of the limitations of conventional proteomic approaches: it offers straightforward target-specific detection, easy operation, high quality data quantitation and excellent assay reproducibility using nanogram levels of sample. As discussed below, this technology has been successfully applied in the dissection of signaling pathways, assessment of proteomic biomarkers, and evaluation of targeted therapies.

## The CNIA technology

**Figure 1:**
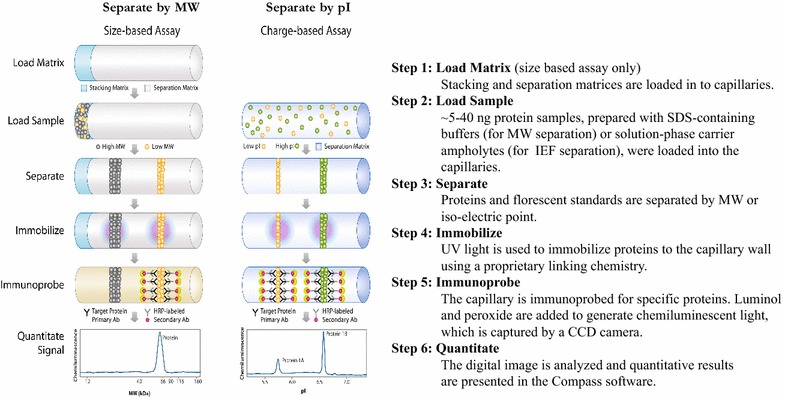
CNIA assay scheme (Courtesy of ProteinSimple).

Capillary nano-immunoassay (also named the Simple Western™ system by the manufacturer, Protein Simple) refers to a capillary-electrophoresis immunoassay system, which offers both size-based and charge-based separation formats. Depending on the process, samples are prepared with SDS-containing buffers (for size-based assay) or solution-phase carrier ampholytes (for charge-based assay) and automatically loaded into small (5 cm, 100 µm internal diameter) capillaries for electrophoretic separation. The CNIA system offer both size-based and charge-based separation formats. Figure 1 shows the assay scheme.

The size-based assay (size-CNIA) separates proteins based on molecular weight. The assay is performed in a similar manner to an SDS-PAGE gel. The protein sample is denatured by heat in the presence of a reducing agent (DTT) and is coated with SDS. The SDS coating gives the proteins in the sample a uniform charge-to-mass ratio. Because the proteins have identical charge-to-mass ratio, each protein’s velocity through the polymer matrix will depend solely on the pores in the polymer matrix and the protein’s radius of gyration, which is proportional to molecular weight.

The charge-based assay (charge-CNIA) differs from a traditional iso-electric focusing (IEF) gel in that the pH gradient is in the solution phase. An “ampholyte gradient” consisting of hundreds of zwitterionic species whose isoelectric points cover a range of pH is added to the sample mixture. When the electric field is applied, the ampholytes arrange themselves according to isoelectric-point (pI) and form a pH gradient throughout the length of the capillary. The proteins and standards migrate through the gradient until they reach their pI, at which point they carry no net charge and cease to migrate. The charge-based assay is also historically referred to as NanoPro™ referring to the company-specific platform or generically as NIA (nanofluidic proteomic immunoassay) assays in the literature.

Electrophoresis in either format is followed by UV light cross-linking of the separated proteins to the capillary wall through proprietary chemistry [18]. The capillaries are then automatically shuttled to an adjacent chamber within the instrument and the capillary lumen is loaded and incubated successively with primary and HRP-tagged secondary antibodies which are flowed through the capillary. The resulting chemiluminescence signal is then detected with a CCD camera, and the digital image is analyzed with Compass software (ProteinSimple, CA, USA). Signal strength is presented as peak area and quantitated. Each sample is prepared with fluorescent pI (for charge-CNIA) or molecular weight (for size-CNIA) markers to calibrate the signal peak profile. Once calibrated, peaks can then be compared between capillaries.

### CNIA assay performance

This novel analysis system offers a number of significant advantages over conventional immunoblot techniques as discussed below.

#### Automation and robust performance

**Figure 2:**
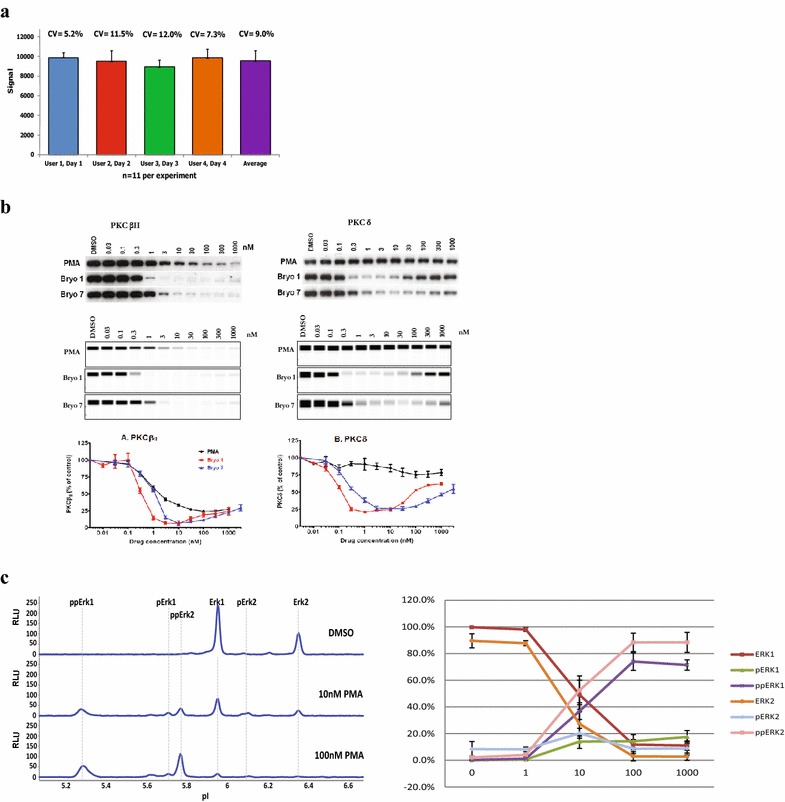
CNIA assay performance at a glance. **a** Assay reproducibility. Size-CNIA data gathered from a CNIA instrument operated by four different users over 4 days. Each run consisted of 11 capillaries analyzing an identical HeLa lysate with an ERK1 antibody using the standard protocol. Instrument software calculated ERK1 peak area in each capillary, and this was used to calculate average and %CV of peak signal (Courtesy of ProteinSimple). **b** A comparison with conventional Western blot and data quantitation. Prostate cancer LNCaP cells were treated with the indicated concentrations of PMA, bryostatin 1, or bryostatin 7 for 24 h. PKCβII and PKCδ were analyzed in total cell lysates by Western blot and size-CNIA using anti-PKCβII and anti-PKCδ antibodies. *Top* and *middle panels* show representative images of conventional Western blot and CNIA respectively. Levels of PKCβII and PKCδ were quantitated from the CNIA data and shown in the *lower panels*. β-actin signals were used as loading controls, and normalized values were expressed relative to that of the DMSO-treated cells. Values represent the mean ± SEM of three independent experiments [38]. **c** ERK isoform responses to PMA treatment detected by Charge-CNIA. RasGRP3 transfected LNCaP cells were treated with indicated concentrations of PMA for 30 min. ERK1 and ERK2 signals were analyzed with charge-CNIA using a pan-ERK antibody. *Left panel* shows the peak profile for the ERK signals, indicating ERK phosphorylation induced by PMA treatment. *Right panel* shows the dose response curve of ERK isoforms to PMA treatment calculated from the CNIA data. Values represent the mean ± SEM of four independent experiments.

By performing all the steps of a charge- or size-based Western blot in a single vessel, the system allows for a level of automation and robust performance that is not possible with conventional immunoblot techniques. The capillaries are ferried between different stations in the instrument for separation/immobilization, antibody incubation, detection, washing and reagent loading. This eliminates all handling during the run and the inconsistencies that can arise from manual intervention. Good intra-, inter-assay reproducibility has been reported with the system [19]. Figure 2a shows an analysis of ERK1 performed by different analysts on different days. An average %CV (coefficient of variation) of less than 10% was observed in this example.

#### Sensitivity, dynamic range, turnaround time, and data quantitation

By taking advantage of the economies of scale in microfluidics, detection sensitivity is much greater than that of other techniques, with results reported on samples corresponding to fewer than 50 cells [18].Typical CNIA runs load nanogram rather than microgram amount of total protein, and have been reported able to precisely measure sub-femtomole amount of target protein [20]. The CCD camera provides 10 times higher dynamic range measurement than film [21]. Analysis linearity over several orders of magnitude has been reported for the system [18, 21–23]. In the 96-capillary throughput analysis platforms (Peggy™, Sally™), one cycle of 12-capillary analysis can be completed in about 2–5 h. A measurement run of up to eight cycles, for a total of 96 samples/analytes can be done in fewer than 24 h, demonstrating a fast assay turn-around time. Recently released instrumentation (Wes™) is capable of running 24 samples simultaneously in under 3 h (see manufacture’s website for more detail on different analysis platforms).

The automated operating system and digital data quantitation allows the CNIA system to provide quantitative and precise measurement of signaling molecules and their activation status [24–30]. The system has demonstrated more accurate and reproducible assessment of protein levels when compared with conventional Western blot analysis and has enabled better correspondence of protein level and function [31–35. In a study of TGF-β-mediated epithelial carcinogenesis [36], Kohn et al. found that Smad3 gene dosage regulates the biological responses to TGF-β. In this study, a mere two-fold reduction in Smad3 was confirmed by precise protein measurements using size-CNIA. This small reduction in protein levels was shown to be sufficient to promote metastasis. Using size-CNIA, Chen et al. developed a method for measuring the absolute amount of endogenous protein at the picogram or sub-picogram level per nanogram of cell lysate [20]. The method provides an approach for precise and accurate assessment of protein levels to correlate with their functions in complex biological settings. The system was also demonstrated to provide quantifiable, consistent and reproducible data that are favorable for clinical translation [37].

With a through-put of 96 sample/analyte combinations in the Peggy™, Sally™ and NanoPro™ platforms of the CNIA system, multiple targets can be profiled simultaneously in one analysis run with low sample consumption. Multiple-analyte analysis in other assays, such as multiplex bead assays and antibody arrays, is constrained by analyte complexity and the potential impact of cross-reactivity. The CNIA system, however, bypasses these obstacles by performing successive cycles of single-analyte analysis each within their separate individual capillaries. This parallel approach thus offers an efficient method of ‘multiplex’ analysis with more accurate and reliable analysis data.

### Assay development and the complementary nature of size-based and charge-based formats

The Size-CNIA assays can be used to detect any protein for which a good antibody is available. Detection of protein modifications is dependent on availability of an antibody specific for that modification. Assay transfer from conventional size-based Western blot to size-CNIA is relatively straight forward for most targets. Based on our experience with developing size-CNIA assays for about two hundred signaling molecule targets, more than 80% of the antibodies that work in conventional size-based Western blot also work in size-CNIA. We observed a good correspondence between conventional Western blot and size-CNIA for the quantitation of PKC isoform down-regulation in U937 cells treated with phorbol esters and bryostatins (Figure 2b) [38]. In the charge-based configuration, using IEF as the separation mode allows for additional levels of information to be obtained. Phosphorylation and other post-translational modification differences in a protein population lead to “charge variants” which can be spatially resolved in IEF. This allows for the simultaneous detection and accurate relative quantitation of these protein variants using a single pan-specific antibody [39, 40]. The identity of the peaks/protein variants has been established experimentally using modification-specific antibodies, site-directed mutagenesis, mass spectrometry and other techniques. See later sections for detailed examples.

The charge-CNIA has been particularly successful in the quantitative analysis of ERK signaling. Using a single detection antibody, ERK1, ERK2 and their phospho-forms can be simultaneously identified, quantitated and compared. Figure 2c shows the IEF profile of ERK isoforms treated with different concentrations of phorbol-12-myristate-13-acetate (PMA). The changes in the dose response curves of individual ERK isoforms (i.e. the dual-phospho-ERK1/2, mono-phospho-ERK1/2, and non-phospho-ERK/1/2) in response to PMA treatment are presented as well. Visualization of the relative abundance of these forms at different concentrations of the drug provides more detail and specific information about signaling molecule activation than is captured by conventional immunoassays.

Using the IEF system to study protein kinase G-I isoform expression in human ovarian cells [41], Fiscus et al. demonstrated that the technology is more than 100 times more sensitive than the conventional Western blot in detecting low abundance PKG-I expressing cancer cells. The technology is also able to resolve and clearly identify the PKG-Iα and PKG-Iβ and the phosphorylation isoforms, which are not distinguishable in Western blot analysis.

The development of charge-CNIA assays may not be as straightforward as the size-CNIA assay and is highly dependent on the specific antibody. In charge-CNIA assay, proteins are analyzed in their native forms without being denatured and reduced as in size-based assays. However, the majority of commercially available antibodies are developed and characterized by recognizing epitopes on denatured proteins. In addition, the data profiles in the charge-CNIA assays, are generally highly complex, requiring multiple control samples and multiple antibodies to be screened before an assay can be confirmed to be specific to the target. Thus, while hundreds of size-CNIA assays have been developed in the past couple of years, the availability of validated charge-CNIA assays is still limited. Nevertheless, when well-validated antibodies are available, charge-CNIA assays are a uniquely powerful tool for generating information that is cumbersome or impossible to generate using other approaches. Antibodies developed for Human Protein Atlas project (http://www.proteinatlas.org/) has been reported may serve as a good source for charge-CNIA assay development [42].

## Applications

The CNIA technology has been demonstrated to be ideal for comprehensive and quantitative protein characterization and profiling of signaling events. It holds great promise as a molecular diagnostic tool to facilitate precision medicine.

### Charge-CNIA, a novel platform for detailed analysis of signaling pathways

As noted above, when there are charge variations among post-translationally-modified isoforms of the target protein, the IEF separation is able to separate and discriminate the different forms, and detect them without using antibodies specific to the modifications [43]. This useful feature of the charge-CNIA has been exploited by investigators to discover novel post-translational modifications (PTM) in a number of signaling pathways and their association with important biological processes.

While there were no phospho-specific Pdx1 antibodies available at the time, Frogne et al. used charge CNIA with an anti-pan-Pdx1 antibody to show that Pdx1, a transcription factor for proper regulation of blood glucose homeostasis and pancreatic beta cell function, underwent post-translational modification in mammalian cells but not in bacterial cells [44]. They further identified that one of the post-translationally-modified variants is unique to mature beta-cells only. Together with alanine scanning and mass spectrometric (MS) analysis, the group determined that serine 61 is the site of the most abundant phosphorylation on Pdx1. In another example, Huang et al. identified a new phosphorylation site on TRAF-interacting protein with an FHA domain (TIFA) that was stimulated by tumor necrosis factor alpha (TNF-α) [45]. In conjunction with MS and in vitro kinase assay analysis, the phosphorylation site was confirmed to be threonine 9. The identification of this novel threonine phosphorylation site provided new insight into the TNF-α mediated signaling pathway, as well as a new functional mechanism for the FHA domain.

Others have used the charge-CNIA to provide novel insights about VEGF induced c-Src phosphorylation changes at pY418 and pY527 [46]. Besides the overall VEGF induced signal change of pY418 and pY527 that is observed in Western blot, the assay also revealed multiple additional phosphorylation isoforms that respond differently to VEGF stimulation, and found that the c-Src pY418 might be a minor fraction of the total phosphorylated c-Src comparing with pY527. The charge-CNIA has also proved to be powerful in the analysis of isoform-specific phosphorylation of AKT, an important target in cancer therapy. Two studies demonstrated that the IEF separation allows simultaneously detection and measurement of all three AKT isoforms with a single pan-reactive antibody, which enabled a more sensitive and accurate comparison of differential regulation roles played by the different isoforms [23, 47].

**Figure 3:**
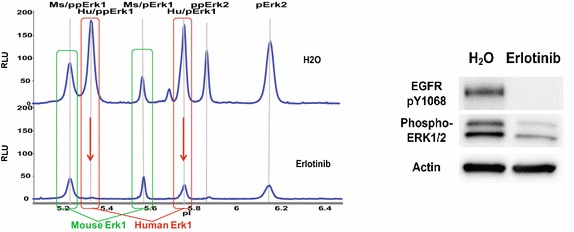
Differential ERK1/2 phosphorylation response to erlotinib treatment in HCC827 xenografts. Mice bearing xenografted tumors of HCC827 human lung adenocarcinoma cells were treated with one dose of water or 100 mg/kg erlotinib and sacrificed 24 h after treatment. *Left panel*, charge-CNIA of ERK phosphorylation in xenograft samples treated with water or with erlotinib. pERK1 and ppERK1 of mouse origin are resolved from the human isoforms and are shown in *green box*. Human pERK1 and ppERK1 are shown in *red box*, and *arrows* indicate that only the human isoform signals decrease with erlotinib treatment. The mouse stromal signal remains unchanged. *Right panel*, conventional Western is not able to detect the differential ERK isoform response between human cancer cells and mouse stroma cells and erroneously implies that ERK signaling by the drug is incomplete in the tumor cells [48].

The high resolution of the charge-CNIA technique can even allow species-specific protein isoforms to be resolved in some cases. In a study of the effect of erlotinib on non-small cell lung cancer (NSCLC) xenograft samples, conventional Western blot analysis indicated that EGFR Y1068 was completely dephosphorylated upon treatment of erlotinib, a tyrosine kinase inhibitor, whereas ERK1/2 appeared to be only partly dephosphorylated (Figure 3). However, using charge-CNIA, human ERK1 isoforms can be differentiated from the mouse isoforms based on their pI differences. Erlotinib was shown to almost completely inhibit ERK phosphorylation in the targeted human xenograft cancer cells but had no effect on ERK phosphorylation in the surrounding mouse stromal cells [48].

Besides phosphorylation, the charge-CNIA assay is also able to detect other PTMs that change the charge of the protein and have functional consequences. Icardi et al. detected a pI shift of STAT3 induced by SIN3 transcription regulator homolog A (Sin3a) and identified K87 acetylation as a negative regulator of STAT3 activity [49]. In a similar approach, Tikhanovich and coworkers identified specific and interactive PTM patterns of phosphorylation, acetylation, methylation and ubiquitination of FOXO3 that were induced by hepatitis C virus, alcohol or the combination of the two, and which alter the activity of this transcription factor [50].

Together, these studies demonstrate that the charge-CNIA system is able to distinguish and detect specific PTM isoforms that conventional Western blot and other immunoassays fail to identify. The technology thus provides a novel platform for in-depth study of signaling pathway mechanisms.

### Quantitative proteomic analysis in limited patient specimens and real-time molecular diagnostics

A key issue in the development of molecular-targeted therapy is the ability to assess whether the drug is hitting the target. Nanogram level sample consumption, digital data processing and quantitation, and proven assay reproducibility make the CNIA system appealing for analysis of clinical specimens to facilitate assessment of pharmacodynamic biomarkers and treatment evaluation. The technology was demonstrated to allow the measurement of stem cell signals from a total of <10,000 cells, which contains a stem cell population of <0.2% [42].

The first CNIA application in analysis of clinical specimens was reported by Fan and coworkers from Stanford in 2009 [22]. In this study, multiple oncoproteins in human tumor samples were precisely quantified using charge-CNIA (NIA). Differential Myc and Bcl2 expression was identified in different types of non-Hodgkin’s lymphoma subtypes. Furthermore, a specific mono-phosphorylated ERK2 signal, which correlated with patient response to tyrosine kinase inhibitor treatment, was identified in chronic myelogenous leukemia (CML) samples.

In a Phase I/II clinical trial, patients with higher risk red blood cell-dependent myelodysplastic syndrome (MDS) were treated with the multi-kinase inhibitor rigosertib [51]. Seetharam and coworkers used charge CNIA to show a direct correlation between changes in the levels of phospho-AKT2 with patient response to drug treatment. CNIA assays were also used to measure rigosertib treatment response in human clinical specimens that are too small to be analyzed by conventional means, such as CD34+ cells from bone marrows and core biopsies from solid tumor tissues [52]. Using a mouse model of head and neck squamous cell carcinoma, Hubbard and coworkers also demonstrated that the system allows molecular diagnostics to be performed longitudinally before, during and after treatment using a minimally invasive technique for specimen collection [53]. These studies indicated that the CNIA system offers a real-time approach to measure target pathway activities and response to treatment in specimen samples that are limited.

The high sensitivity of the system has also enabled the profiling of signaling molecules in other samples of limited availability [48, 54]. ERK activation in aberrant crypt foci (ACF) was measured and compared to the adjacent normal colonic muscosa in micro-dissected human clinical samples containing as little as 16 ng protein from fewer than 200 cells [55]. Other examples that exploit the high sensitivity of this technique include the analysis of target molecule response in FACS-sorted blast crisis leukemia stem cells derived from bone marrow of tyrosine kinase inhibitor-treated mice [56], label-retaining cancer stem cells derived from human hepatocellular carcinoma [57], and assessing the pS962 PTRC/CD45 level in CD34+ cells from CML patients [58].

### Development of pharmacodynamic and predictive biomarkers

**Figure 4:**
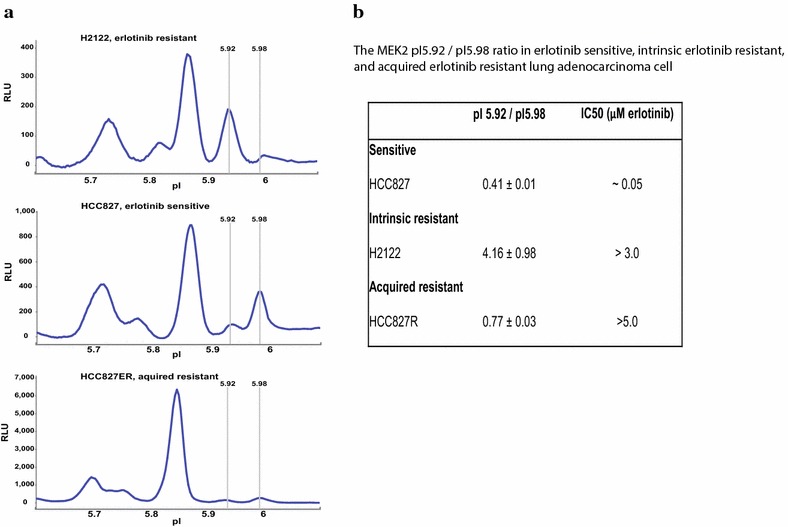
MEK2 peaks correlated with erlotinib sensitivity in NSCLC cells. **a** NSCLC cells with different sensitivity to erlotinib were profiled for MEK2 activation by charge-CNIA. A higher MEK2 peak with a pI of 5.92 (“R”) is observed in the intrinsically erlotinib resistant H2122 cells, while a higher MEK2 peak with a pI of 5.98 (“S”) is observed in erlotinib sensitive HCC827 cells. The MEK2 pI 5.98 “S” signal was also decreased after the HCC827 cells were cultured in escalating concentrations of erlotinib which led to acquired resistance to the drug. **b** Quantitation of relative R and S peaks in the three cell lines [48].

Although this application has not been as fully exploited, the CNIA technology shows promise in the development of biomarkers that predict response to therapy [22, 51, 59, 60]. In a study of the dynamic phosphorylation status of signaling molecules in NSCLC cells treated with EGFR tyrosine kinase and MEK inhibitors, a specific on-target MEK response pattern to a MEK inhibitor was identified, which was not detectable by conventional Western blot [48]. The authors also identified a MEK2 signal that may be associated with NSCLC cell sensitivity to the EGFR inhibitor erlotinib (Figure 4). Using a charge-CNIA, Beurlet showed that treatment with the BCL-2 inhibitor ABT-7373 restored wild-type MEK phosphorylation patterns in mice with acute myelogenous leukemia, associated with extended lifespan and increased survival. [61].

### Quality assessment in development and manufacture of biological drugs

The CNIA technology has important industrial applications as well. The CNIA platforms have been reported to be the choice for product quality control analysis based on it being a high-throughput and automated analysis system, and its proven assay precision, accuracy and sensitivity. Michels et al. demonstrated that the charge-CNIA offers specificity, speed and sensitivity advantages over an imaged capillary isoelectric focusing platform when used to analyze charge heterogeneity of monoclonal antibody products in early stage process development [62]. Rustandi and colleagues applied size-CNIA to protein expression and identity in vaccine development. They reported that the technology increased the efficiency of analytical testing and could more effectively guide process development [63]. The group also developed a new improved method for detection of residual BSA to meet quality control requirements in vaccine production [64].

## A comparison of CNIA to other immunoassay technologies

Immunoassay-based analytical technologies have a long history and have been widely used for target-specific protein detection and quantification from discovery research to clinical studies. The following narrative offers a comparison of the CNIA system with other major immunoassay platforms. The advantages and challenges for each technology are highlighted in Table 1.

**Table 1:** The pros and cons of different immunoassay technologies

	Pros	Cons
Size-CNIA	Nanogram sample loading, good reproducibility, quantitative data, medium throughput for multi-sample or multi-target analysis, straight forward assay transfer from conventional Western blot, automated operation, easy protocol standardization	Limited matrix options for high resolution analysis on small and big MW proteins
Charge-CNIA	Nanogram sample loading, quantitative data, good reproducibility, medium throughput for multi-sample or multi-target analysis, distinguish and detect isoform variations or PTMs with pan-reactive antibody, automated operation, easy protocol standardization	Often need to test multiple antibodies for a target, limited ampholyte options for low and high pI protein analysis, peak identity determination is challenging
Conventional Western blot	Has been top choice for studying protein isoform variations, post-translational modifications	Microgram sample loading, poor data quantitation and reproducibility, low throughput, tedious manual processing steps, challenge to transfer assay from discovery research for clinical application
IHC	Provide information about protein localization within the cell, most used immunoassay for clinical tissue sample analysis	Poor data quantitation and reproducibility, data interpretation is subjective, difficult in protocol standardization
ELISA	Has been gold standard for protein concentration measurement, quantitative data, good reproducibility	Microgram sample loading, need isoform specific antibody, stringent antibody evaluation for specificity, relative long assay development
Multiplexed bead assays	Multiplex, high throughput, small sample consumption, automation	Potential cross-reactivity issues, stringent antibody evaluation for specificity, long assay development process, expensive reagents and consumables
Antibody array	Multiplex, high throughput, small sample consumption, automation	Potential cross-reactivity issues, stringent antibody evaluation for specificity, long assay development process
RPPA	Multiplex, high throughput, ng sample loading, automation	Stringent antibody evaluation for specificity, long assay development process, high cost when perform low sample number multi-target analysis

### ELISA (Enzyme-linked immunosorbent assay) and multiplexed bead assays

ELISA is the most widely used and highly validated method for protein concentration measurement. It remains a gold standard immunoassay for drug development programs. A digital ELISA approach has been reported that detects serum proteins at sub-femtomolar concentrations [65]. Multiplex ELISA assays have been developed in recent years to quantify multiple proteins in complex biological samples [66, 67]. Applications of multiplexed assays have been mainly in biological fluid samples, e.g. serum and plasma. Applications in complex bio-specimens, such as tissue or whole cell lysates, are limited due to confounding matrix effects. There are also challenges when optimizing analysis conditions simultaneously for multiple proteins with different affinities for the detecting antibodies. Due to antibody cross-reactivity issues, most commercial kits are limited to fewer than 30 targets in a single measurement [68, 69]. Traditional ELISA assays usually require large amount of samples, and the lead time for development is long (usually several months to more than 1 year) [13]. In addition, though evidence has suggested the importance of protein isoforms in disease progress, ELISA and multiplex-bead assays are limited in accessing this information due to the lack of isoform specific antibodies to differentiate different modifications and/or protein variants [16, 70].

### Conventional Western blot

Combining protein separations with target-specific antibody probing, Western blot has been the top choice for studying protein levels and post-translational modifications, due to the low cost and simplicity of the technology. However, the conventional Western blot technology suffers from large sample and reagent usage, time and labor commitment, semi-quantitative data, poor reproducibility, and low throughput. Currently, Western blot is mainly used in discovery research and encounters difficulties in further development for pre-clinical and clinical applications [69, 71].

### Protein arrays

Protein arrays are somewhat analogous to chip-based transcriptional profiling for analysis of proteins and their functioning status. There are two types of protein arrays, antibody arrays and reverse-phase protein arrays (RPPA). The antibody arrays involve immobilizing well-characterized antibodies on a solid support which are then used for detecting multiple target proteins or PTMs in single sample. Labeled protein mixtures are applied to the array followed by immuno-analysis. In contract, multiple tissue or cell lysates, instead of antibodies, are used to construct RPPAs., The arrays are then probed by target-specific antibodies, thus allowing multiple samples to be queried simultaneously for a single target. Like CNIA protein arrays offer multiplexed protein profiling in low sample volumes. Protein arrays have been successfully applied in clinical proteomics, biomarker identification, therapeutic monitoring, biomarker discovery, and evaluation of pharmaceutical targets. [72–74]. Quantitative proteomic profiling of signaling pathways from surgical and micro-dissected tumor specimens and fine-needle aspirates were shown to be promising for guiding potential therapeutic intervention strategies [73, 75, 76].

The antibody array and RPPA face several technical challenges compared with CNIA. Since the platforms cannot identify nonspecific binding of the antibodies to other proteins, so rigorous antibody validation procedures are required to assure absolute specificity [10, 15, 77], Moreover, additional specific antibodies are needed to detect protein isoform variants [68]. As in multiplexed bead assays, antibody array-based protein measurements are usually limited to 30–50 targets due to antibody cross-reactivity [78]. RPPA has the advantage over CNIA that hundreds of samples can be analyzed in a single slide for a single target, with the detection sensitivities of 1000-5000 molecules/spot [77]. However, the technology can be expensive when only a small number of samples are evaluated across many targets.

### Immunohistochemistry

Immunohistochemistry (IHC) detects endogenous proteins in situ and thus provides spatial information about heterogeneous expression of proteins in their natural environment within a tissue that is not provided by bulk biochemical analysis techniques such as CNIA [79]. Traditional IHC analysis is the most widely used immunoassay for clinical tissue sample analysis and has demonstrated the power of biomarker analysis to drive clinical decisions. However, IHC data are usually semi-quantitative and lack of reproducibility making the approach difficult to use when quantitative information is important, for example, when assessing pharmacodynamic biomarker responses to targeted therapies. Since IHC data assessment primarily relies on the individual pathologist’s experience, it is difficult to standardize procedures when data interpretation is subjective [80].

## Further improvements of the CNIA technology

As discussed in this manuscript and elsewhere, the CNIA technology has shown considerable promise for quantitative sensitive proteomic analysis. For the future development and translational application of the technology, some improvements are needed.

### Expanding the range of target detection with good resolution

To accommodate the analysis of a broad range of protein size and charge, new reagents and assay conditions will need to be developed. In the size-based analysis platform, current reagents and assay conditions are optimized for the separation of proteins with MW between 12 and 230 kDa. Based on our experience, when proteins with molecular weights lower than 20 kD, variation in estimated molecular weight or low signals were observed for some proteins. In addition, although a new kit for 66–440 kD protein analysis has been released (see the ProteinSimple website for product detail), more extensive validation of the kit performance is desired. In the IEF system, good assay resolution and reproducibility are observed between pI 4–8. While this covers most proteins, reduced resolution and lower assay reproducibility can be observed with especially low or high pI targets.

### Improving signal-to-noise ratio in size-CNIA

In general, straight-forward assay transfer and good data consistency with conventional Western blot assays has been observed with the size-CNIA assays. Occasionally, high background has been observed in the size-CNIA assays. This phenomenon is mostly primary antibody-dependent. It may be solved by additional blocking of the capillaries, increasing the antibody dilution factor, or switching to an alternative primary antibody. We have seen a higher baseline noise in the higher-sensitivity size-CNIA analysis mode that uses the newer Peggy Sue™ technology. Though this analysis mode offers more than five-fold increase in signal, in our experience, the higher noise level seen with some targets can jeopardize data quantification and assay reproducibility. Also, the Peggy Sue analysis mode has been observed to be sensitive to climate factors, such as low humidity in the winter months. This analysis mode needs further development to restore the robustness that is an important positive attribute of the CNIA assays.

### Peak identification for charge-CNIA

The comprehensive in-depth information obtained for signaling molecule activation by the IEF system is exciting. However, it also raises the challenge of determining the identities of the peak components revealed by the system. For a complete understanding of the complicated peak profiles and their correlations to the function of signaling molecules, other technologies will need to be employed. In cases where isoform- and PTM-directed antibodies are available along with knockout cells and/or mutant constructs, peak identity has been determined [23, 46, 47, 50]. Mass spectrometry (MS) peak identification protocols have also been developed and used for identification of previously unknown phosphorylation sites on their targets in the CNIA system [44, 45, 81]. For broader application, peak ID protocols will need to be developed for more complex PTM patterns.

## Integrating the CNIA technology with complementary proteomics approaches

It is likely that the specific advantages of many of the different technologies can be usefully combined in ways that exploit the specific strengths of each. There are reports of using RPPA and antibody arrays as an initial discovery screening to identify predictive assays that are later transferred to CNIA analysis with reduced complexity for potential clinical applications [82, 83]. With the further development and improvement of the CNIA system, it is expected that complementary and supplementary applications of this new technology with other immunoassay platforms will emerge.

Like other immunoassays, antibody performance and availability in many circumstance may limited the applications of CNIA assays. In the past decade, the field of MS-based proteomics has grown tremendously. MS-based multiple reaction monitoring (MRM) assays provide targeted, quantitative and multiplexed analysis of proteins and respective PTM isoforms without the requirement for an antibody [84, 85]. The MS technology has demonstrated to be a powerful discovery platform. Though more complicated sample processing and data analysis are involved with MS assays, and more sophisticated expertise is required to operate the system, MRM–MS assays can be rapidly configured for verification of protein biomarker candidates before further moving to clinical-grade immunoassays. An in-depth discussion of the MS technology is beyond the scope of this review. Interested readers may refer to Libeler’s recent review [84] about MRM for more information. As mentioned in the previous session that MS protocols have been used as a strategy to detect the detail identity of PTM isoforms revealed by the charge-CNIA assays [44, 45, 81]. Integrating the different analysis methods will allow a more comprehensive study of proteins and their functioning mechanisms and will help translate the discovery research data into clinical practice.

## Conclusions

As reviewed here, the CNIA system provides comprehensive and quantitative analysis for protein characterization and profiling of signaling events. The automated system offers easy operation, precise and accurate measurement of proteins and their post-translational modifications and a fast turn-around time. The capillary platform allows functional proteomic analysis to be performed with limited sample, such as from stem cells, primary cells, fine needle aspirates, micro-dissection samples and other patient specimens. Good intra-assay, inter-run and inter-person data reproducibility have been observed with the system, which enables assay standardization across multiple testing sites.

With the further validation of CNIA assays and development of the technology, it is expected that a growing number of applications in both discovery research and clinical practice for signaling pathway dissection, proteomic biomarker assessment, and targeted therapy evaluation. The high reproducibility and data precision of the technique mean the technology can be readily adapted for use in CLIA-certified settings. Complemented with other genome, transcriptome and additional protein analysis technologies, the CNIA technology shows great promise for developing pathway specific diagnostics for better treatment group stratification and acceleration of drug development and disease management.

## Abbreviations

CNIA: capillary nano-immunoassay
PTM: post-translational modification
IEF: iso-electric focusing
Size-CNIA: size-based capillary nano-immunoassay
Charge-CNIA: charge-based capillary nano-immunoassay
ELISA: enzyme-linked immunosorbent assay
%CV: coefficient of variation
IHC: immunohistochemistry
RPPA: reverse-phase protein array
MS: mass spectrometry
MRM: multiple reaction monitoring
